# *Cis*-regulatory elements: systematic identification and horticultural applications

**DOI:** 10.1007/s42994-025-00237-0

**Published:** 2025-08-08

**Authors:** Tian Li, Wen Zeng, Fangjie Zhu, Peitao Lü

**Affiliations:** 1 Haixia Institute of Science and Technology, Fujian Provincial Key Laboratory of Haixia Applied Plant Systems Biology, College of Horticulture, National Engineering Research Center of JUNCAO, Fujian Agriculture and Forestry University, Fuzhou, 350002 China; 2 National Key Laboratory of Tropical Crop Breeding, Institute of Tropical Bioscience and Biotechnology & Sanya Research Institute, Chinese Academy of Tropical Agricultural Sciences, Sanya, 572024 China

**Keywords:** Breeding, *Cis*-regulatory element, DNA-binding specificity, Epigenetics, Gene regulatory network, Horticultural crops

## Abstract

*Cis*-regulatory elements (CREs) are the genetic DNA fragments bound by transcription factors (TFs). CREs function as molecular switches that precisely modulate the dosage and spatiotemporal patterns of gene expression. The systematic identification of CREs not only facilitates the annotation of the functional non-coding genome but also provides essential insights into the architecture of gene regulatory networks and sheds light on an accurate selection of the target sites for genetic engineering of crops. In this review, we summarize the current high-throughput methodologies used for identifying CREs, illustrate the associations between CREs and agronomic traits in horticultural crops, and discuss how CREs can be exploited to facilitate crop breeding.

## Introduction

Higher plants and animals typically contain tens to hundreds of different cell types (Rood et al. 2025). Although their morphologies and physiological functions are diverse, all cells in the same organism share an identical genome and the same set of genes. It is thus the dosages of genes—that is, the expression levels of individual genes—that explain the differences between cells. The expression of a gene is determined by its associated *cis*-regulatory elements, such as enhancers, promoters, and silencers. Due to low mapping resolution, CREs have sometimes been localized to only a broad region defined as the gene promoter (e.g., 1–3 kb upstream of the transcription start site). However, CREs are much smaller, specific sequences located within this region, where they are bound by TFs that regulate gene expression. Therefore, in this review, we define CREs as TF binding sites (TFBSs): 6- to 20-bp stretches of DNA that are recognized by sequence-specific TFs (Khamis et al. 2018).

CREs have been extensively studied since the emergence of molecular biology. Following the development of cloning methods and Sanger sequencing (first-generation sequencing), CREs were inserted in front of reporter genes to examine their effects on transcription (Angel et al. 1987). Because the binding of CREs by TFs is required for their regulatory functions, biochemical assays, such as electrophoretic mobility shift assays (EMSAs) (Hellman et al. 2007) and DNA footprinting (Hampshire et al. 2007), were developed to investigate whether a CRE is bound by a TF. These methods examine only one or a few CRE sequences at a time. Although they provide important insights into the regulatory relationships between a few genes, these low-throughput methods are unable to decipher the overwhelming complexity of gene regulatory networks (GRNs).

The development of second-generation (next-generation) sequencing techniques has greatly boosted sequencing yields. High-throughput methods such as ChIP-seq, DNase-seq, and Hi-C enable the systematic identification of CREs across the entire genome. Consortium programs, such as ENCODE (Moore et al. 2020), ROADMAP (Kundaje et al. 2015), and 4D-Nucleome (Dekker et al. 2017), have standardized the protocols for these techniques and generated comprehensive datasets to identify and describe mammalian CREs. The datasets allow for an in-depth dissection of the architecture of mammalian GRNs (Gerstein et al. 2012). Corresponding efforts to profile plant CREs have lagged but have seen significant advances in recent years (Fu et al. 2022; O’Malley et al. 2016; Wang et al. 2023; Xie et al. 2021). To facilitate further research and the application of plant CREs, in this review, we summarize the available methodologies for systematically profiling CREs based on distinct mechanisms. Then, focusing on horticultural crops, we discuss how CREs regulate important agronomic traits and can be exploited to facilitate crop breeding.

## SYSTEMATIC IDENTIFICATION OF *CIS*-REGULATORY ELEMENTS

CREs can be systematically identified through direct and indirect approaches. Direct approaches involve identifying DNA sequences that are recognized and bound by TFs. Indirect approaches involve locating CREs based on their downstream effects after being bound by TFs (e.g., chromatin opening, histone modification, and transcriptional activation). Here we classify the available techniques for CRE identification and discuss their advantages and disadvantages.

### CRE identification based on TF binding sites

**Fig. 1:**
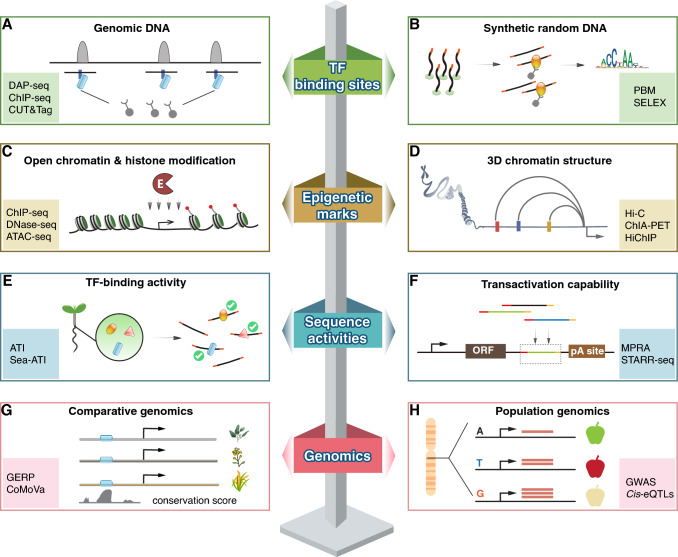
Representative methodologies for the systematic identification of CREs. First, CREs for a single TF can be identified by profiling the TF’s binding sites. The input library may utilize genomic DNA to directly locate the TF’s regulatory targets (**A**) or synthetic random DNA to derive accurate TF binding models (**B**). Second, although at lower resolution, genome-wide CREs can be inferred by profiling open chromatin regions and histone modifications (**C**), and regulatory relationships between CREs and their target genes can be explored by analyzing the 3D chromatin structure (**D**). Third, assessing either the TF-binding activity (**E**) or the transactivation activity (**F**) of DNA sequences can identify CREs with high confidence. Fourth, comparative genomics (**G**) and population genomics (**H**) can identify functional non-coding sequences (including CREs) that are conserved or associated with different phenotypes

In essence, CREs are TF binding sites located in gene promoters, enhancers, silencers, insulators, or sometimes coding regions (Doni Jayavelu et al. 2020; Gotea et al. 2010; Whitfield et al. 2012). Therefore, identifying TFBSs is a straightforward way to detect CREs in the genome at high resolution. Classical methods, such as EMSA (Hellman et al. 2007) and footprinting (Hampshire et al. 2007), can only identify a few sequences bound by a TF. By contrast, second-generation sequencing provides the sequences for all TFBSs in the genome simultaneously (Fig. 1A).

DNA affinity purification-sequencing (DAP-seq), a process that involves incubating genomic DNA with tagged recombinant TFs, enriches all genomic fragments containing CREs of the target TF (Bartlett et al. 2017). To date, the largest plant CRE dataset obtained by DAP-seq is a genome-wide binding atlas of 529 Arabidopsis (*Arabidopsis thaliana*) TFs (O’Malley et al. 2016). DAP-seq has also been used to systematically profile the TFBSs for maize (*Zea mays*) auxin response factors (Galli et al. 2018) and for wheat (*Triticum aestivum*) and soybean (*Glycine max*) TFs (Jiao et al. 2024; Zhang et al. 2022). Modified versions of DAP-seq, including double DNA affinity purification-sequencing (dDAP-seq) (Li et al. 2023) and sequential DNA affinity purification sequencing (seq-DAP-seq) (Lai et al. 2020), have been developed to identify genomic TFBSs of TF heterodimers. For non-model organisms where tagged-TF vectors are not readily available, biotin-DAP-seq offers a preferable solution by introducing the biotin tag during translation (Baumgart et al. 2021). To understand how CREs evolve across phylogenetically relevant species, multiDAP pools barcoded gDNA from multiple species in the pull-down (with the TF of interest), and thereby parallelly reveals CREs in all these species with a single assay (Baumgart et al. 2021, 2025).

Although easily performed with high throughput, DAP-seq uses naked DNA that lacks its cellular chromatin context, such as chromatin accessibility (Perino et al. 2016), histone modifications (Xin et al. 2018), and other cofactors (Wang et al. 2021c), all of which influence TF–DNA binding in vivo. Moreover, recombinant TFs expressed in vitro do not contain all post-translational modifications. These limitations can be overcome by using chromatin immunoprecipitation followed by sequencing (ChIP-seq). ChIP-seq uses anti-TF antibodies to immunoprecipitate genomic sequences bound by endogenous TFs (Johnson et al. 2007) to examine TFBSs in their natural chromatin context. However, ChIP-seq has several limitations: (1) the requirement for high-specificity antibodies; (2) potential epitope masking during formaldehyde crosslinking (Kidder et al. 2011); (3) non-specific binding of irrelevant chromatin fragments; and (4) the need for a large number of input cells (10^5^–10^7^).

Several improvements to the original ChIP-seq have been made to address these issues. For limitation (1), semi-in vivo ChIP-seq uses epitope-tagged TFs to eliminate the need for specific antibodies, enabling the rapid, low-cost, scalable profiling of genomic CREs (Tu et al. 2020). For limitation (2), natural ChIP (N-ChIP) does not require formaldehyde fixation and instead operates at low temperature to prevent dissociation (Kasinathan et al. 2014). N-ChIP is better for analyzing histones than TF–DNA interactions because the latter interactions are weaker and may dissociate (Schmidl et al. 2015). For limitation (3), non-specific binding can be reduced using chromatin endogenous cleavage coupled with high-throughput sequencing (ChEC-seq), which fuses the target TF to micrococcal nuclease (MNase), through which ChEC-seq selectively releases TFBS fragments while keeping other parts of the chromatin intact and insoluble (Gera et al. 2024; Zentner et al. 2015). To avoid the need for sequence engineering of the target TFs, cleavage under targets and release using nuclease (CUT&RUN) uses antibody-coupled MNase to release DNA fragments around the TFBSs; this technique features a high signal-to-noise (S/N) ratio (Skene et al. 2017). For limitation (4), the Tn5 tagmentation-based method cleavage under targets and tagmentation (CUT&Tag) has been developed to increase the efficiency of adaptor ligation and reduce the operating time (Ma et al. 2020): this technique can be used with as few as 100–1000 cells (Skene et al. 2018) and even a single cell (Kaya-Okur et al. 2019). For plant materials, the classic ChIP methods involve a nuclei isolation step (Kaufmann et al. 2010), which leads to > 90% material loss because the cell wall traps large amounts of nuclei in the debris. The nuclei isolation step is not required in enhanced ChIP (eChIP), which can be applied to as little as 0.01 g of plant tissue (Zhao et al. 2020b). In advanced ChIP (aChIP), the S/N ratio of eChIP is further increased by removing irrelevant cytosolic components before immunoprecipitation (Zhang et al. 2024). However, despite the availability of ChIP-seq and all its derivatives, detecting TFBSs in vivo remains challenging for plants because high-quality antibodies are not yet available for most plant TFs (Park 2009).

DAP-seq, ChIP-seq, and their derivatives are powerful techniques for identifying TFBSs in the genome, but these assays often fail to yield binding motifs for the TFs examined. For instance, motifs were successfully derived for only about 50% of DAP-seq libraries (Zhang et al. 2022). Moreover, because DAP-seq and ChIP-seq typically yield 10^3^–10^4^ peaks from a limited number of TFBS sequences, it is difficult to build unbiased models for high-information content motifs such as those of TF dimers (Li et al. 2025). Therefore, to comprehensively profile the biochemical specificity of TFs, binding assays can be performed using randomized DNA instead of genomic DNA as the input library (Fig. 1B). The randomized input library can contain up to 10^16^ potential sites for TF binding, whereas a typical genome only offers 10^8^–10^10^ sites. The protein binding microarray (PBM) approach immobilizes all possible 8-mers or 11-mers onto a microarray and measures their affinities to the target TF (Berger et al. 2009; Godoy et al. 2011). PBM assays can be performed at high-throughput (Berger et al. 2006) and have generated binding models for more than 100 plant TFs (Franco-Zorrilla et al. 2014; Lambert et al. 2019).

Although PBM provides accurate models that describe the biochemical affinity of a TF, this technique cannot capture the binding of distantly spaced TF dimers due to the length limit of the k-mer used (Jolma et al. 2013). To overcome this issue, systematic evolution of ligands by exponential enrichment (SELEX) relies on synthetic random DNA sequences that are up to 200 bp and has been successfully used to capture the dimeric binding of TFs spaced up to 80 bp apart (Zhu et al. 2018). SELEX can be performed at either low-throughput (EMSA-based) (Slattery et al. 2011; Smaczniak et al. 2017; Van Mourik et al. 2023) or high-throughput (affinity purification-based) (Fan et al. 2020; Jolma et al. 2013; Li et al. 2025; Wang et al. 2021b). Many techniques have been developed based on SELEX. Consecutive affinity purification SELEX (CAP-SELEX) examines the specificity of TF heterodimers; this technique successfully generated 9400 TF-TF–DNA interaction profiles (Jolma et al. 2015). Methyl-SELEX and EpiSELEX-seq provide TF binding models in the presence of 5-methylcytosines in the CG context (Kribelbauer et al. 2017; Yin et al. 2017) or the CG, CHG, and CHH contexts (Jiang et al. 2025; Morgunova et al. 2025). Nucleosome consecutive affinity purification SELEX (NCAP-SELEX) explores TF binding on the nucleosome (Zhu et al. 2018). Although the original SELEX method has been extensively utilized to study plant TFs (Käppel et al. 2021; Mao et al. 2024; Smaczniak et al. 2017; Van Mourik et al. 2023), including a recent high-throughput study (Li et al. 2025), other SELEX-based techniques have yet to be established for plants. Unlike the PBM approach, SELEX is performed in multiple rounds to increase the signal strength. This results in an exponential enrichment that exaggerates the affinity differences between strong and weak TFBSs. The computational models NRLB (Rastogi et al. 2018), BEESEM (Ruan et al. 2017), and ProBound (Rube et al. 2022) have been developed to correct such biases and derive biophysical models from SELEX data.

Overall, the above methods profile TFBSs in either the genome or synthetic random ligands, depicting all genomic targets of a TF or its accurate specificity landscape. However, the most important drawback of these methods is that they only investigate TFBSs for one TF at a time, whereas in cells, transcriptional regulation is a coordinated process.

### CRE identification based on epigenetic marks

Genomic regions enriched with active CREs are frequently characterized by specific epigenetic marks. Among all epigenetic marks, histone marks exhibit the greatest variety. Histone marks are covalent modifications that usually occur in histone tails, including methylation, phosphorylation, acetylation, ubiquitylation, sumoylation (Millán-Zambrano et al. 2022), and lactylation (Zhang et al. 2019a). Histone marks define chromatin states and regulate gene expression (Candela-Ferre et al. 2024; Murgas et al. 2025). The genome-wide distribution of histone marks is routinely examined by ChIP-seq and its derivatives. Such analyses also shed light on genome-wide CREs because a portion of histone marks are closely associated with CREs (Fig. 1C). For example, in animals, active promoters are marked by H3K4me3 and enhancers by H3K9ac and H3K27ac (Heintzman et al. 2007; Shlyueva et al. 2014), while H3K27me3-rich genomic regions can function as silencers (Cai et al. 2021). In plants, H3K4me3 also characterizes active promoters, but different from animals, H3K27me3 not only marks silenced promoters but also extends into the associated gene bodies (Xiao et al. 2016). H3K4me1 and H3K27ac are not related to enhancers in plants: H3K4me1 modifies the transcribed regions of gene bodies, and H3K27ac primarily marks promoter-proximal regions (Xiao et al. 2016; Yan et al. 2019).

In addition to histone marks, active genomic CREs are often associated with an open chromatin state because the binding of TFs to CREs can displace the associated nucleosomes. Open chromatin co-localizes with both active promoters and enhancers. Consequently, profiling open chromatin defines all types of CREs within a single experiment and is generally preferred over profiling histone marks. In formaldehyde-assisted isolation of regulatory elements (FAIRE-seq), free DNA fragments (from open chromatin) are separated from nucleosome-associated fragments based on their different phase partitioning behaviors (Simon et al. 2012). The workflow of FAIRE-seq is straightforward, but the S/N ratio is low (Davie et al. 2015). The most commonly used methods to probe open chromatin are transposase-accessible chromatin using sequencing (ATAC-seq) (Grandi et al. 2022) and DNase I-hypersensitive site sequencing (DNase-seq) (Han et al. 2022). Both techniques rely on an enzyme (Tn5 or DNase I) to selectively release DNA fragments from open chromatin. ATAC-seq is more popular than DNase-seq because it adds adaptors through tagmentation, which improves ligation efficiency and decreases the required sample size and operating time. Micrococcal nuclease sequencing (MNase-seq) is typically used to profile nucleosome-occupied regions that are located “opposite” open chromatin (Zhang et al. 2018). However, slight digestion by MNase can also release DNA fragments from the open chromatin (Zhao et al. 2020a); this finding led to the development of MNase hypersensitivity sequencing (MH-seq). Although these enzyme-based methods feature a higher S/N ratio than FAIRE, the enzymes also have nucleotide preferences that should be considered when quantitatively modeling the strengths of CREs: MNase prefers AT-rich DNA (Chung et al. 2010); DNase I cleavage is sensitive to the width of the DNA minor groove, which in turn depends on nucleotide composition and DNA methylation (Lazarovici et al. 2013); and Tn5 functions as a dimer to bind to palindromic Tn5 motifs (Li et al. 2019a).

Analyzing 3D chromatin structure also provides unique insights into CREs. At low resolution, chromatin can be divided into A (active) and B (inactive) compartments (Lieberman-Aiden et al. 2009). Nearly all active promoters and enhancers localize to the A compartment (Harris et al. 2023). High-resolution 3D architecture further reveals the interactions between CREs and their target genes (Jung et al. 2019) (Fig. 1D). Such information is particularly useful for large genomes, as assigning distal enhancers to their regulatory targets can be difficult with large genomes (Ricci et al. 2019). Although CRE–gene pairs can also be assigned by performing correlation analysis between chromatin openness and gene expression, hundreds of sequencing libraries are required for such analysis (Zhu et al. 2024). By contrast, the assignment of CRE–gene pairs based on 3D chromatin structure only requires a single library. A series of proximity ligation-based chromosome conformation capture (3C) techniques is available to probe 3D chromatin structure. Hi-C (Akgol Oksuz et al. 2021; Lieberman-Aiden et al. 2009) and Micro-C (Hsieh et al. 2016, 2015) capture all possible chromatin interactions across the genome (Liu et al. 2024). Hi-C uses restriction enzymes such as *Hin*dIII, *Dde*I, and *Dpn*II for chromatin fragmentation, but Micro-C uses MNase and achieves higher, nucleosome-level resolution. Hi-C, a technique based on the non-sequence-specific nuclease DNase I, has also been developed (DNase Hi-C) to improve resolution (Ramani et al. 2016). Hi-C and Micro-C can be directly applied to plants with small genomes to identify all CRE–gene associations along with other chromatin interactions. However, as the number of chromatin interactions (and therefore the required sequencing depth) increases exponentially with increasing chromatin size, it is more economical to analyze large genomes by focusing only on CRE–gene interactions. This can be achieved by performing immunoprecipitation to select chromatin interactions near a target protein, such as a TF or a modified histone. Based on this rationale, chromatin interaction analysis by paired-end tag (ChIA-PET) (Wang et al. 2021a) and Hi-C followed by chromatin immunoprecipitation (HiChIP) (Mumbach et al. 2016) have been developed. HiChIP has fewer false positives and requires fewer cells than ChIA-PET because in this technique, chromatin contacts are fixed in the nucleus (in situ) before cell lysis, and library construction is performed using on-bead Tn5 tagmentation (Mumbach et al. 2016). Because open chromatin better represents all active CREs than histone marks, open chromatin enrichment and network Hi-C (OCEAN-C), which combines the workflows of FAIRE-seq and Hi-C, were also developed (Li et al. 2018).

The annotation of CREs based on epigenetic marks has been widely explored in ENCODE projects for plants. This work has led to the generation of comprehensive epigenetic maps (Zhao et al. 2020b; Lü et al. 2018; Li et al. 2019b; Liao et al. 2022; Zhang et al. 2023a; Luo et al. 2025a), yielding 10^4^ to 10^5^ CRE regions in different species. However, CREs annotated with epigenetic marks have limited resolution because the peaks of these marks often range from hundreds to thousands of base pairs in width.

### CRE identification based on sequence activities

Functional CREs differ from other genomic sequences in that CRE sequences bind to their cognate TFs and regulate transcription. Inspired by this finding, high-throughput methods have been established to comprehensively identify all active CREs (cistrome) in a tissue of interest. Active TF identification (ATI) assays have been established to target the TF-binding activities of CRE sequences (Fig. 1E) (Wei et al. 2018; Wen et al. 2023). In these assays, a complex pool of DNA sequences is incubated with nuclear proteins (including TFs), and EMSA is used to select shifted bands harboring CRE sequences that can bind to nuclear TFs. Both random DNA and genomic DNA sequences can be used in ATI, providing insights into TF binding models and genomic CRE sites, respectively. ATI was originally developed for animal cells and tissues (Wei et al. 2018) and includes a nuclei isolation step that is inefficient in plants. The modified Sea-ATI (sequential extraction assisted active TF identification) protocol lacks a nuclei isolation step and can be used with as little as 50 mg of plant tissue (Wen et al. 2023). Both ATI and Sea-ATI have identified motif models not reported before, suggesting that eukaryotic TFs have yet to be fully characterized.

Targeting the transcriptional regulatory activities of CREs (Fig. 1F), high-throughput reporter assays have been developed to assess the CRE activities for 10^5^ to 10^6^ sequences in parallel. In massively parallel reporter assays (MPRAs), the examined sequences are inserted upstream of a reporter gene and therefore cannot be transcribed, necessitating the introduction of short barcodes near the end of the reporter gene to keep track of sequence identity (Kheradpour et al. 2013; Melnikov et al. 2012; Patwardhan et al. 2012). In self-transcribing active regulatory regions sequencing (STARR-seq), the candidate CRE sequences are placed into the transcribed 3'UTR of a reporter gene, thereby coupling the CRE activity of a sequence to the abundance of its self-transcription (Arnold et al. 2013; Liu et al. 2017). This improvement increases the throughput of the assay and allows CRE activity to be assessed on a genome-wide scale. However, both MPRAs and STARR-seq rely on transient transfection, which limits their application to a few established cell types. The use of lentivirus-based MPRAs (lentiMPRAs) has expanded the scope of application to include more cell types and even organoids (Agarwal et al. 2025). Notably, the CREs identified in MPRAs overlapped poorly with the active CREs that bind to TFs (CREs identified by ATI) (Sahu et al. 2022), presumably because most reporter assays overlook CREs with weak or repressive activities (Das et al. 2023). To fill this gap, Silencer screening STARR-seq (Ss-STARR-seq) was recently developed (Zhu et al. 2025b; a). High-throughput reporter assays are also powerful tools for identifying plant CREs (Jores et al. 2021, 2020; Sun et al. 2019; Tan et al. 2023; Tian et al. 2022), but few plant tissues are suitable for this technique. To date, only leaves and protoplasts have been successfully used for MPRAs and STARR-seq.

The use of sequence-activity-based methods to identify CREs has multiple advantages. First, these methods identify CREs recognized by all active TFs in a single assay and are therefore more efficient than TFBS-based methods. Second, sequence-activity-based methods yield CREs that are highly likely to be functional, unlike epigenetic-mark-based methods. However, a major deficiency of sequence-activity-based methods is that the sources of the identified CREs are unclear: their cognate TFs are not self-evident from CRE sequences or motifs. Additional transcriptome and proteome data can help clarify the associations between CREs and TFs.

### CRE identification based on genomics

Although CREs reside in non-coding genomic sequences, they represent functional genomic elements that are maintained by weak purifying selection (Steige et al. 2017). Therefore, CREs can also be identified by phylogenetic footprinting, a technique for profiling evolutionarily conserved non-coding sequences (CNSs) across multiple genomes (Fig. 1G) (Polychronopoulos et al. 2017). Phylogenetic footprinting complements simple motif-matching analysis to reduce false positives. Most phylogenetic footprinting studies performed to date have involved conservation analysis based on genome-wide sequence alignment, such as the genomic evolutionary rate profiling (GERP) score (Cooper et al. 2005; Davydov et al. 2010), phastCons score (Siepel et al. 2005), phyloP score (Pollard et al. 2010), and SiPhy (Garber et al. 2009). Alternatively, sequence alignment can focus only on promoters, such as STAG-CNS (Lai et al. 2017). However, conserved genomic regions are much shorter in plants than in vertebrates. This reduces the detection power of alignment-based methods, especially for multiple species separated by large evolutionary distances (Zemlyanskaya et al. 2021). To address this issue, the alignment-free tool CNEFinder was developed to identify CREs based on *k*-mer matches between genomic sequences (Ayad et al. 2018). CoMoVa is also available for detecting conserved small degenerate sequences in known motifs over broad evolutionary distances (Lieberman-Lazarovich et al. 2019). CREs defined by phylogenetic footprinting are significantly enriched in regulatory regions and have been functionally linked to important agronomic traits in model species, such as *Arabidopsis*, maize*,* and cucurbit crops (Luo et al. 2025b; Song et al. 2021; Van de Velde et al. 2016; Xin et al. 2024; Yocca et al. 2021).

In plants with large genomes, many CREs reside in enhancers that are located up to tens of kilobase pairs away from the target gene. In this case, superimposing epigenetic marks (e.g., chromatin accessibility, histone modification, and DNA methylation) onto the results of phylogenetic footprinting can facilitate the localization of distal active chromatin and help pinpoint the CREs within these regions (Lu et al. 2019; Maher et al. 2018; Ricci et al. 2019). Including epigenetic marks when predicting proximal CREs can further reduce the rate of false positives.

Additionally, population genomics offers the opportunity to identify CREs by associating genomic variants with different phenotypes (Fig. 1H). These phenotypes can be either biochemical or physiological. Associations with physiological phenotypes are examined in genome-wide association studies (GWAS). Over 90% of GWAS variants fall into non-coding regions of the genome. These variants are potential CREs, as they tend to accumulate in regulatory genomic regions (Maurano et al. 2012) and frequently disrupt TFBSs (Musunuru et al. 2010). Associations with biochemical phenotypes are assessed during the analysis of expression quantitative trait loci (eQTLs). This technique measures genomic sequences and transcriptomes for tens to hundreds of individuals and identifies genomic variants that are correlated with gene expression levels. The eQTLs within 1 Mb of the target gene are called *cis*-eQTLs (Võsa et al. 2021), the majority of which are CREs (Gong et al. 2018). Integrating GWAS variants and *cis-*eQTLs more reliably captures CREs (Patel et al. 2021). For example, a combined GWAS and *cis*-eQTL analysis in *Paeonia suffruticosa* identified CREs controlling petal and stamen number (Peng et al. 2023). Models from eQTL analysis are also utilized in transcriptome-wide association studies (TWAS) to establish gene–trait associations (Gamazon et al. 2015; Gusev et al. 2016).

### CRE identification in single cells

Traditional CRE identification methods rely on bulk tissue analysis. However, biological tissues are inherently heterogeneous, comprising multiple cell types and cells in various states. This heterogeneity extends to the cistrome and transcriptome, as different cell types exhibit distinct active CREs and gene expression profiles. Bulk-phase methods obscure such heterogeneity by averaging signals across all cells, potentially masking crucial cell-type-specific regulatory dynamics. Recent advances in single-cell technologies have provided various solutions to overcome these limitations.

Single-cell ChIP-seq (scChIP-seq) (Grosselin et al. 2019; Rotem et al. 2015) and single-cell CUT&Tag (scCUT&Tag) (Bartosovic et al. 2021) can be used to identify CREs based on TFBSs. To identify CREs based on epigenetic marks, histone modifications can be assessed using scChIP-seq (Rotem et al. 2015) and scCUT&Tag (Bartosovic et al. 2021); chromatin accessibility can be measured by single-cell DNase-seq (scDNase-seq) (Jin et al. 2015), single-cell ATAC-seq (scATAC-seq) (Fang et al. 2021; Yuan et al. 2022), and single-cell MNase-seq (scMNase-seq) (Lai et al. 2018). Three-dimensional chromatin structure can be profiled using single-cell Hi-C (Rappoport et al. 2023) and Droplet Hi-C (Chang et al. 2024). To identify CREs based on sequence activity, the single-cell massively parallel reporter assay (scMPRA) was recently developed to investigate the transcriptional effect of CREs (Zhao et al. 2023).

For bulk-phase assays, chromosome conformation capture (3C) methodologies are preferable for establishing the regulatory relationships between CREs and their target genes. This is because the alternative approach—analyzing the co-regulation of CRE accessibility and gene expression—requires hundreds of ATAC-seq and RNA-seq libraries. By contrast, for single-cell assays, the paired measurements of chromatin accessibility and gene expression across a population of cells provide sufficient data for exploring CRE–gene relationships. This involves single-cell combinatorial indexing (sci-CAR) (Cao et al. 2018) and single-cell chromatin accessibility and transcriptome sequencing (scCAT-seq) (Liu et al. 2019), which effectively integrates scATAC-seq and scRNA-seq into a single protocol, allowing for the construction of GRNs within a specific tissue.

Single-cell technologies have been increasingly used in plant research. To examine transcriptional regulation and facilitate CRE identification, scRNA-seq (Cantó-Pastor et al. 2024; Efroni et al. 2016), single-cell Hi-C (Zhou et al. 2019), and scATAC-seq (Dorrity et al. 2021; Marand et al. 2021, 2025) were successfully performed in *Arabidopsis*, tomato (*Solanum lycopersicum*), rice (*Oryza sativa*), and maize. Despite these advances, many state-of-the-art single-cell technologies have yet to be adapted for plants, as the rigid plant cell wall can hinder the effective isolation of individual cells. Additionally, protoplast isolation requires tailored optimization across species and tissues (Rhaman et al. 2024). Consequently, most single-cell measurements performed to date have focused on soft tissues (e.g., young leaves and roots). For tissues recalcitrant to single-cell isolation, single-nucleus RNA-seq (snRNA-seq) can be employed as an alternative (Habib et al. 2016). However, an enormous challenge remains in that many CRE identification methods require high-integrality nuclei (e.g., scATAC-seq), which are difficult to isolate from tissues with thick cell walls and abundant secondary metabolites.

Single-cell techniques are ideally complemented by spatially resolved techniques to localize single-cell profiles within a specific tissue context. Current spatially resolved techniques include spatial-ATAC-seq (Deng et al. 2022b), which profiles open chromatin regions, and spatial-CUT&Tag (Deng et al. 2022a), which maps TFBSs and histone modifications. To construct GRNs, spatial-ATAC–RNA-seq and spatial-CUT&Tag–RNA-seq can be combined to profile chromatin accessibility and histone modifications genome-wide alongside the transcriptome within the same tissue section at near-single-cell resolution (Zhang et al. 2023b), providing a comprehensive view of regulatory interactions in a spatial context.

### CRE databases for plants

The high-throughput methods described above have generated an enormous amount of data about CREs in different plant species. Accordingly, CRE databases have been developed to facilitate data access and provide online analytical tools (Chow et al. 2023; Huo et al. 2024).

The earliest databases of plant CREs were constructed using TFBS-based CREs. PlantCARE (Lescot et al. 2002) and PLACE (Higo et al. 1999) were established before the emergence of high-throughput techniques and stored the consensuses of TFs and the verified TFBS sequences in promoters. The stored TFBS sequences were then used to predict CREs in the user-inputted sequence. Following the development of high-throughput methods, TFBS information is now stored as position weight matrices (PWM), and information about individual TFBSs in the promoters of target genes is stored as genome-wide TFBS peaks. The updated versions of TFBS-based CREs are now systematically curated in the following databases: PWM matrices in PlantPAN (Chow et al. 2024), PlantTFDB (Jin et al. 2017), JASPAR (Rauluseviciute et al. 2024), CIS-BP (Weirauch et al. 2014), TRANSFAC (Matys et al. 2006), UniBind (Puig et al. 2021), FootprintDB (Contreras-Moreira et al. 2016), and MethMotif (Dyer et al. 2024); and genome-wide TFBS peaks in ReMap (Hammal et al. 2022), ChIP-Hub (Fu et al. 2022), and SoyGRN (Jiao et al. 2024).

Epigenetic-mark-based CREs are derived from chromatin accessibility data (e.g., DNase-seq, MNase-seq, ATAC-seq), histone modification data (e.g., ChIP-seq, CUT & Tag), and 3D chromatin structure data (e.g., Hi-C, Micro-C). Chromatin accessibility and histone modification data are systematically curated in databases such as PlantRegMap (Tian et al. 2020), Plant Regulomics (Ran et al. 2020), ChIP-Hub (Fu et al. 2022), PlantCADB (Ding et al. 2023), ChIPBase (Huang et al. 2023), PCSD (Liu et al. 2018), EGDB (Luo et al. 2025a), AraENCODE (Wang et al. 2023), and RiceENCODE (Xie et al. 2021). Furthermore, 3D chromatin structure data are included in ChromLoops (Zhou et al. 2023).

Few databases contain information about sequence-activity-based CREs. MPRAVarDB (Jin et al. 2024) and RAEdb (Cai et al. 2019) include data from MPRA and STARR-seq assays performed in mammalian cell lines. This type of database has yet to be developed for plants.

## *CIS*-Regulatory elements in horticultural crops

**Fig. 2:**
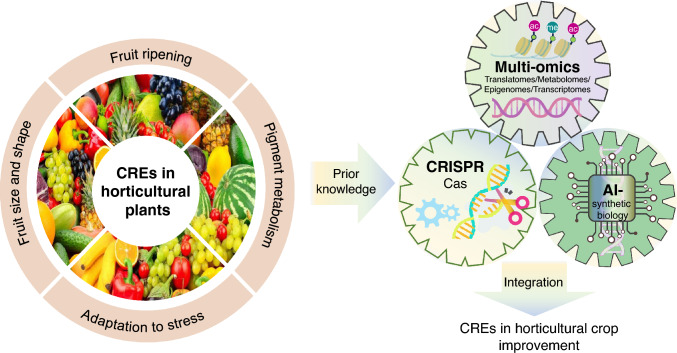
*Cis*-regulatory elements in horticultural crops. Horticultural research has demonstrated that CREs play crucial roles in regulating various agronomic traits. The high-throughput identification of CREs, coupled with AI-assisted design and CRISPR/Cas-mediated editing, should significantly enhance the efficiency and precision of horticultural breeding

In horticultural crops, CREs are involved in the regulatory networks of all physiological processes, such as growth, environmental adaptation, and the development of quality traits. During domestication and crop improvement, natural selection and artificial breeding have optimized not only the coding regions of genes but also the associated CREs that regulate their expression. The selection and engineering of CREs in horticultural crops have shaped many vital agronomic traits, such as fruit quality, color, and stress resistance (Fig. 2). Here, we discuss the CREs responsible for several traits and address the potential applications of CREs in breeding.

### CREs that regulate fruit ripening

The fruit is the organ with the highest economic value in most horticultural crops. The dynamic GRNs and associated CREs that function during fleshy fruit ripening have been extensively examined. In tomato, the promoter of the MADS-box TF gene *RIN* contains an EIN3-binding site; this CRE is crucial for the ethylene-mediated regulation of ripening-related genes. Genome-wide chromatin accessibility and DNA methylation analyses have revealed the hypermethylation of this CRE at the mature green stage of fruit development. In the breaker stage, SlDML2-mediated epigenetic reprogramming induces DNA demethylation, facilitating chromatin relaxation, EIN3 protein binding, and subsequent *RIN* activation (Lang et al. 2017; Liu et al. 2015; Lü et al. 2018; Zhong et al. 2013). Similarly, hypermethylation of a 286-bp region upstream of *SlSPL-CNR* in the tomato *Cnr* mutant blocks the binding of RIN to its CRE CArG, disrupting the normal ripening process and leading to the failure to soften and redden (Martel et al. 2011).

The dual-loop ripening system in banana (*Musa acuminata*) exemplifies hierarchical regulation through TF–CRE interactions. The EIN3-binding element in the *MaNAP1* promoter activates the primary circuit, while reciprocal binding between MaMADS1 and MaNAP1 to each other’s promoter establishes a dual-loop secondary network (Lü et al. 2018). Notably, the presence of an EIN3-binding motif in the *MaMADS1* promoter enables a direct interaction between MaEIN3 and the MaNAP1-MaMADS1 module. This module coordinates cell wall-related gene expression and TFs through CRE recognition, forming a ripening regulatory network (Li et al. 2024a). In apple (*Malus domestica*), the promoter of *MdNAC18.1*, which encodes a master regulator of ripening, harbors a 61-bp repeat containing NAC-recognition motifs. MdNAC18.1 binds to its promoter to establish autoinhibition, enabling the precise control of gene expression during ripening (Zhang et al. 2025). Furthermore, the *MdEXP-A1* promoter, which encodes a protein that governs cell wall loosening, contains a 1166-bp transposable element (TE-1166) with dual NAC-binding ACGT motifs. Deleting TE-1166 reduced promoter activity, increased fruit firmness, and disrupted MdNAC1 binding, confirming the role of this transposable element in the direct transcriptional activation of *MdEXP-A1* (Su et al. 2025).

### CREs that modulate fruit size and shape

CREs that regulate fruit size and shape have also been explored. During the domestication of cultivated tomato varieties from wild tomato (*Solanum pimpinellifolium*), SNPs in the promoter regions of *FW2.2* (*SlCNR*) and *FW3.2* (*SlKLUH*) were selected that reduce the expression of these genes, leading to enlarged fruits (Chakrabarti et al. 2013; Frary et al. 2000). Similarly, two SNPs in the 15-bp inhibitory CRE downstream of *SlWUS* disrupt the binding of the TF AGAMOUS, enabling sustained *SlWUS* expression during late fruit development. This leads to an increased locule number and fruit size (Muños et al. 2011). CRISPR-mediated editing of conserved CREs near *NAC1* and *EXT-like* genes significantly reduced fruit size in cucumber (*Cucumis sativus*) (Xin et al. 2024).

Regarding fruit shape, a 31-kb deletion upstream of the tomato *SlOFP20* gene downregulates its expression, which in turn releases the inhibition of the OVATE pathway and enhances longitudinal cell division and fruit elongation (Wu et al. 2018). An alternative mechanism involves the *SUN* gene. A *Copia*-like retrotransposon (Rider) relocated *SUN* from chromosome 10 to chromosome 7, positioning it downstream of the enhancer-like promoter of *DEFL1* (a defensin gene). This genomic rearrangement drives the ectopic high expression of *SUN* in fruits and promotes longitudinal cell expansion, leading to an elongated fruit morphology (Jiang et al. 2009; Xiao et al. 2008). These findings underscore the critical roles of CREs in shaping fruit morphology.

### CREs that regulate pigment metabolism

Anthocyanins are water-soluble flavonoid pigments in plants that give rise to a variety of hues ranging from red/pink to purple/blue. Therefore, the CREs that regulate anthocyanin biosynthesis have attracted much interest. *MdMYB1* is responsible for anthocyanin accumulation in apple fruit. The *MdMYB1* promoter in red-skinned varieties contains an LTR retrotransposon (*redTE*) with enhancer activity. The insertion of *redTE* increases *MdMYB1* expression under low-light or cold conditions by recruiting environmental stress-responsive TFs. DNA methylation of *redTE* also varies across cultivars, adding a further layer to the regulation of apple skin color (Zhang et al. 2019b). A parallel mechanism governs the red pigmentation of apple flesh: tandem 23-bp repeats in the *MdMYB10* promoter have established a transcriptional autoactivation circuit, allowing sustained anthocyanin biosynthesis (Espley et al. 2009). In mandarin orange (*Citrus reticulata*), a MITE transposon inserted into the *CCD4b* promoter contains CREs that upregulate *CCD4b* expression. This insertion drives C30 carotenoid production, resulting in the red coloration of the peel (Zheng et al. 2019). Similarly, blood orange (*Citrus sinensis*) exhibits cold-dependent anthocyanin biosynthesis mediated by a *Copia*-like retrotransposon (*Tcs1*) adjacent to *Ruby*. Cold-inducible *Tcs1* expression activates *Ruby*, linking pigmentation to environmental conditions (Butelli et al. 2012). Conversely, white-berry phenotypes in grapevine (*Vitis vinifera*) arise from a *Gret1* retrotransposon insertion in the *VvMYBA1* promoter, which decreases the expression of this TF gene, thereby reducing anthocyanin accumulation (Kobayashi et al. 2004).

Collectively, these studies have established MYB TFs as central regulators of pigment metabolism and emphasized the importance of CREs in their promoter regions in controlling fruit color. Transposon-derived CREs, methylation-sensitive CREs, and repetitive autoregulatory CREs exemplify different evolutionary strategies that fine-tune fruit pigmentation based on developmental and environmental cues.

### CREs that mediate plant adaptation to abiotic stress

Although the mechanisms of environmental adaptation in horticultural crops remain largely unclear, chromatin accessibility has recently been shown to be dynamic at CREs near stress-responsive genes. In cold-sensitive banana cultivars, WRKY binding sites (W-box) are enriched in the promoters and active enhancers of cold-induced genes. Chilling triggers WRKY TFs to bind to these CRE clusters and remotely activate browning-related gene networks through chromatin looping (Zhu et al. 2023). These findings delineate a genome-wide mechanism underlying cold-induced peel browning through CRE clustering. The adaptation of banana to high temperatures involves a different mechanism. MaMYB60 directly activates chlorophyll catabolism genes via the MYB recognition sequences in their promoters. Thermal stress induces MaBAH1-mediated ubiquitination and degradation of MaMYB60, thereby suppressing the expression of chlorophyll catabolism genes to retain green pigmentation (Wei et al. 2023). The proteolytic regulation of upstream TFs of CREs represents a novel thermoadaptive process in fruits.

Cross-species analyses have provided further examples of CRE-mediated stress responses. In ‘Nanguo’ pear (*Pyrus ussuriensis* Maxim.), the R2R3-MYB regulators PuMYB21/54 bind to MBS motifs in the *PuPLDβ1* promoter to activate membrane lipid peroxidation, accelerating peel browning under oxidative stress (Sun et al. 2020). Grape berries exposed to light show enhanced cuticular wax biosynthesis and reduced postharvest deterioration. This response is achieved through the binding of VvHYH and VvGATA24 to the G-box/GATA CREs in the promoters of *VvTPS12* and *VvHMGR2* (Yang et al. 2024). These findings collectively demonstrate how CRE plasticity enables the transcriptional reprogramming of horticultural crops under abiotic stress and offer molecular targets for enhancing crop resilience and postharvest quality. The coordinated actions of stress-inducible TFs through conserved CREs represent an evolutionary framework for environmental adaptation in fleshy fruits.

### CREs that drive horticultural crop improvement

CREs have also become popular targets in crop breeding (Song et al. 2022). Compared to targeting the coding regions of genes, engineering CREs has three major advantages: (1) dosage control—the expression levels or spatiotemporal expression patterns of genes can be modulated without gene knockout-induced lethality; (2) reduced pleiotropy—targeting tissue-specific enhancers/repressors can minimize off-target developmental effects; (3) evolutionary agility—CREs can undergo rapid evolution due to their localization in the non-coding regions of genes, which are under relaxed purifying selection. These attributes make *cis*-regulatory engineering a transformative approach for overcoming pleiotropic constraints and achieving trait modularity in crop improvement (Rodríguez Leal et al. 2017).

After functional CREs are characterized, CRISPR/Cas-mediated genome editing can be used for the precise engineering of the CREs to achieve desirable agronomic traits. A typical strategy is to modify the CREs within native promoters. In *Citrus* species (*C. paradisi* and *C. sinensis*), CRISPR-mediated editing of the *LOB1* promoter was used to modify the CRE that is recognized by the PthA4 pathogen effector and to generate canker-resistant plants by disrupting bacterial virulence targets (Jia et al. 2019, 2016; Peng et al. 2017). Similarly, multiplex CRISPR editing of the promoters of development-related genes in tomato (e.g., *CLV3*, *WUS*, *SP*) created an allelic series with quantitative effects on yield-related traits, illustrating the power of *cis*-regulatory mutagenesis for trait optimization (Rodríguez Leal et al. 2017). Alternatively, synthetic CREs can be inserted into the promoters of target genes to realize a programmable transcriptional pattern. In a pioneering study in tomato, heat-shock response elements were inserted into the open chromatin regions of the *LIN5* (*CWIN*) promoter, enabling heat-inducible modulation of sucrose partitioning. This spatial–temporal regulation of source-sink dynamics provides a climate-resilient strategy for yield optimization (Lou et al. 2025). Such approaches exemplify how synthetic CREs can be used to rewire transcriptional networks to address emerging agricultural challenges.

The integration of artificial intelligence (AI) and synthetic biology is redefining CRE engineering. This allows for the de novo design of synthetic CREs with customized regulatory logic, making it possible to design tissue-specific enhancers or stress-inducible promoters (Gosai et al. 2024; Li et al. 2024b, c; Zhang et al. 2023c). Synthetic biology turns computational design into reality by providing modular assembly frameworks. AI-designed CREs can be combinatorially integrated into synthetic promoter architectures, enabling the engineering of metabolic pathways and stacked trait expression. In crop engineering, such systems could be used for multiplex optimization, such as enhancing disease resistance using pathogen-responsive promoters while maintaining yields through growth-phase-specific expression circuits.

## Conclusions

Given the critical roles of CREs in GRNs, researchers have focused for decades on answering the following questions: What are CREs (biochemical specificity)? Where are they located (genomic TFBSs)? How do they function (transcriptional and physiological effects)? The advent of high-throughput methodologies has led to an exponential increase in the volume of publicly available CRE data. The expansion of CRE datasets is expected to accelerate further, but the accumulated data already provides substantial insights into the three questions mentioned above, especially the first two.

Innovations in data analysis will be crucial for advancing our understanding of CRE grammar. AI and deep learning-based models offer unparalleled opportunities for classification and generation tasks, such as the identification and de novo design of CREs. However, the complexity of neural networks—comprising numerous units and parameters—often fragments the predictive power of an intact model. For instance, filters in a convolutional layer typically capture only partial motifs, and individual parameters within a network lack direct biophysical interpretability. Therefore, the development of less complex yet carefully designed machine learning models will be crucial for extracting biological insights and deriving thermodynamic parameters for CREs.

In this review, we summarized TFBS-based, epigenetics-based, activity-based, and genomics-based approaches to CRE identification. A combination of these methods is expected to facilitate the systematic identification of CREs that bind to TFs, regulate the deposition of epigenetic marks, control transcription, and correlate with phenotypic observations. Although these methods offer a comprehensive set of CRE targets for crop breeding, their implementation is currently hindered by the time-consuming processes of gene editing and phenotyping. A straightforward solution is to develop more efficient multiplex editing platforms tailored to specific crop species. Alternatively, the phenotypic effects of target CREs could be evaluated in model plants with shorter life cycles. Expanding the collection of natural germplasm will also enhance CRE polymorphism at target genomic loci.

Natural variation in CREs and the “mutation-and-selection” strategy have shaped the domestication trajectories of horticultural crops. In future breeding efforts, the precise design and targeted editing of CREs will enable “predictive trait engineering”, facilitating the development of horticultural crops with enhanced climate resilience and improved nutritional value, thereby contributing to the sustainability of global agriculture.

## Data Availability

Data sharing is not applicable to this article as no datasets were generated or analyzed during the current study.
